# Generation and Characterization of Single Chain Variable Fragment against Alpha-Enolase of *Candida albicans*

**DOI:** 10.3390/ijms21082903

**Published:** 2020-04-21

**Authors:** Sy-Jye Leu, Yu-Ching Lee, Chi-Hsin Lee, Po-Yen Liao, Chen-Wei Chiang, Chieh-Ming Yang, Ching-Hua Su, Tsong-Yih Ou, Ko-Jiunn Liu, Hsiu-Jung Lo, Bor-Yu Tsai, Yi-Yuan Yang

**Affiliations:** 1 Department of Microbiology and Immunology, School of Medicine, College of Medicine, Taipei Medical University, Taipei 11031, Taiwan; cmbsycl@tmu.edu.tw (S.-J.-L.); curator@tmu.edu.tw (C.-H.S.); 2 Graduate Institute of Medical Sciences, College of Medicine, Taipei Medical University, Taipei 11031, Taiwan; keviandy121@gmail.com (P.-Y.L.); b114096001@tmu.edu.tw (C.-W.C.); m120103029@tmu.edu.tw (C.-M.Y.); 3 Center for Reproductive Medicine and Sciences, Taipei Medical University, Taipei 11031, Taiwan; 4 The Center of Translational Medicine, Taipei Medical University, Taipei 11031, Taiwan; ycl@tmu.edu.tw; 5 School of Medical Laboratory Science and Biotechnology, College of Medical Science and Technology, Taipei Medical University, Taipei 11031, Taiwan; chihsine@msn.com (C.-H.L.); kojiunn@nhri.org.tw (K.-J.L.); 6 Division of Infectious Diseases, Department of Internal Medicine, School of Medicine, College of Medicine, Wan Fang Hospital, Taipei Medical University, Taipei 11696, Taiwan; 93023@w.tmu.edu.tw; 7 National Institute of Cancer Research, National Health Research Institutes, Tainan 70456, Taiwan; 8 National Institute of Infectious Diseases and Vaccinology, National Health Research Institutes, Tainan 70456, Taiwan; hjlo@nhri.org.tw; 9 School of Dentistry, China Medical University, Taichung 40402, Taiwan; 10 Navi Bio-Therapeutics Inc., Taipei 10351, Taiwan; boryutsai@navibio.com.tw; 11 Core Laboratory of Antibody Generation and Research, Taipei Medical University, Taipei 11031, Taiwan; 12 Ph.D. Program in Medical Biotechnology, College of Medical Science and Technology, Taipei Medical University, Taipei 11031, Taiwan

**Keywords:** *C. albicans*, alpha–enolase, phage display technology, single chain variable fragment

## Abstract

*Candida albicans* (*C. albicans*) is an opportunistic human pathogen responsible for approximately a half of clinical candidemia. The emerging *Candida* spp. with resistance to azoles is a major challenge in clinic, suggesting an urgent demand for new drugs and therapeutic strategies. Alpha–enolase (Eno1) is a multifunctional protein and represents an important marker for invasive candidiasis. Thus, *C. albicans* Eno1 (CaEno1) is believed to be an important target for the development of therapeutic agents and antibody drugs. Recombinant CaEno1 (rCaEno1) was first used to immunize chickens. Subsequently, we used phage display technology to construct two single chain variable fragment (scFv) antibody libraries. A novel biopanning procedure was carried out to screen anti-rCaEno1 scFv antibodies, whose specificities were further characterized. The polyclonal IgY antibodies showed binding to rCaEno1 and native CaEno1. A dominant scFv (CaS1) and its properties were further characterized. CaS1 attenuated the growth of *C. albicans* and inhibited the binding of CaEno1 to plasminogen. Animal studies showed that CaS1 prolonged the survival rate of mice and zebrafish with candidiasis. The fungal burden in kidney and spleen, as well as level of inflammatory cytokines were significantly reduced in CaS1-treated mice. These results suggest CaS1 has potential of being immunotherapeutic drug against *C. albicans* infections.

## 1. Introduction

Candida diseases are often chronic, difficult to treat, and cause high mortality and morbidity, regardless of extensive anti-fungal therapy. *Candida* spp. are the third leading microorganisms of infections in intensive care units globally [1,2]. Previous studies have indicated that *Candida* spp. are ranked fifth among hospital-acquired pathogens and fourth among bloodstream infection pathogens [3,4]. The diagnosis of invasive candidiasis is difficult due to the lack of specific clinical features and the low sensitivity of blood culture for the isolation of *Candida* spp. Blood culture remains the gold standard for the diagnosis of candidemia [5]. *Candida albicans* is the most important fungal pathogen causing human diseases [6,7]. Amphotericin (AmB) is a gold standard of antifungal treatment for fungal infections, but the severe side effects of this drug restrict its clinical application [8]. The widespread and prolonged use of azoles has led to the rapid development of the phenomenon of multidrug resistance (MDR) [4,9]. Several reports have shown that the incidence of pathogenic strains with resistance to fluconazole has increased dramatically during the last two decades [10,11,12,13].

Enolase is present in all tissues and organisms capable of glycolysis or fermentation. In mammals, α-enolase (Eno1) is expressed ubiquitously in various types of tissues and has been found to be involved in several biological and pathophysiological processes [14,15]. Eno1 is ubiquitously expressed in the cytosol and on the cell surface as a plasminogen-binding receptor [16,17,18,19]. *C. albicans* Eno1 (*Ca*Eno1)-null mutants exhibit altered drug susceptibility, hyphal formation, and virulence [20], strongly suggesting that the expression of *Ca*Eno1 is critical for the cell growth of *C. albicans*. Mutations in the *Ca*Eno1 exhibit inhibitory activity on cell growth [21]. The extracellular Eno of *C. albicans* mediated the colonization of small intestine, which activity could be suppressed by anti-Eno antibodies [22].

Altogether, these studies indicate that the CaEno1 molecule may be a suitable target for the development of therapeutic drugs to cure infectious and cancerous diseases. In the present study, a scFv monoclonal antibody (CaS1) was isolated by phage display technology, which recognized the recombinant CaEno1 (rCaEno1) and CaEno1 expressed by *C. albicans* and *C. tropicalis*. Moreover, CaS1 attenuated the growth and plasminogen binding activity of *C. albicans*. More importantly, the CaS1 prolonged survival time and reduced the fungal burden and inflammatory cytokine levels of mice in an in vivo candidiasis model. Therefore, CaS1 could be a starting point for further development of *C. albicans* -specific therapies.

## 2. Results

### 2.1. Analysis of Anti-CaEno1 IgY Antibodies

**Figure 1:**
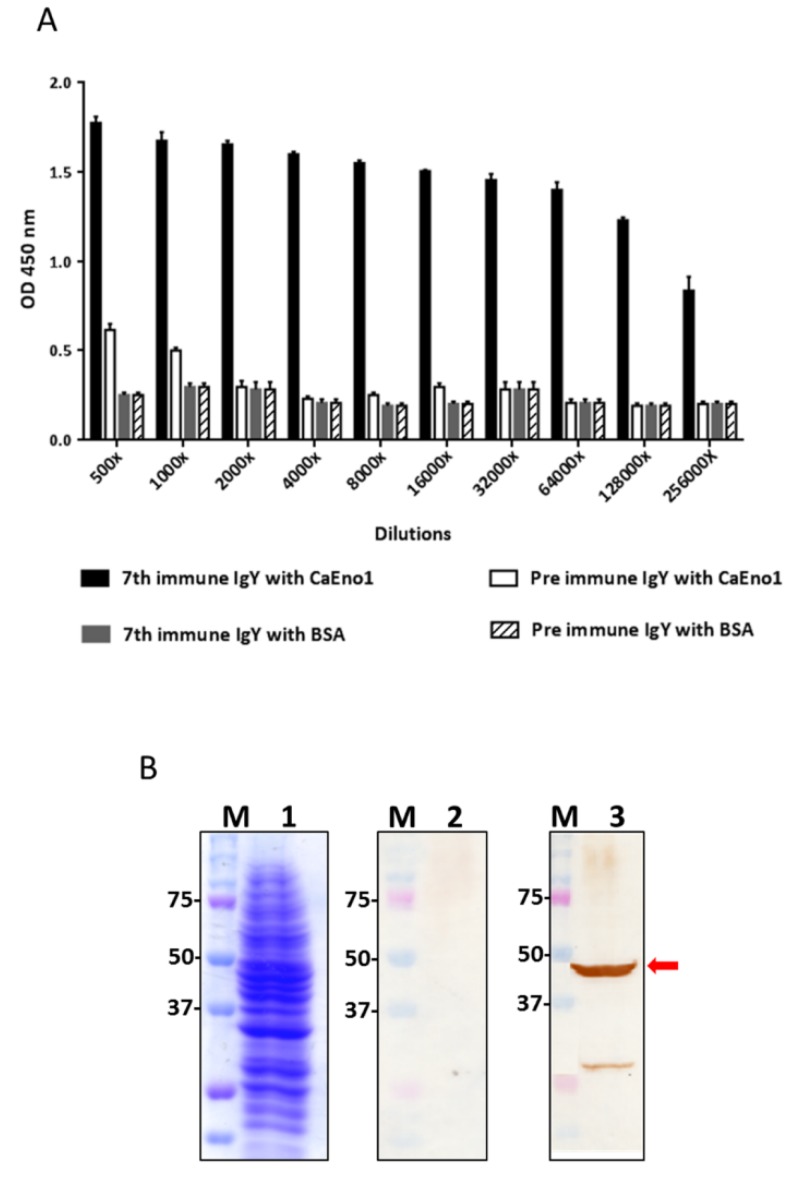
Humoral antibody response in chickens. The purified recombinant *C. albicans* alpha-enolase 1 (rCaEno1) protein and BSA (negative control) were coated onto ELISA plates. A series of diluted polyclonal IgY antibodies from pre-immunized or 7th-immunized chickens were evaluated for their binding activities (**A**). Total cell lysates of *C. albicans* were visualized by SDS-PAGE (**B**, lane 1). Membranes immobilized with lysate proteins were incubated with IgY antibodies (1:5000) from pre-immunized (**B**, lane 2) or serum after the 7th-immunization round (**B**, lane 3), followed by HRP-labeled donkey anti-chicken IgY (1:3000).

The humoral immune response in chickens was analyzed by ELISA and Western blotting (Figure 1A,B). As shown in Figure 1A, compared to those from preimmunized chickens, the partially purified polyclonal IgY antibodies from chickens (Supplementary Figure S1) after the 7th immunization exhibited specific binding to CaEno1 but not to BSA. Notably, anti-CaEno1 IgY antibodies at 128,000× dilution showed significant absorbance at an optical density at 450 nm. The data in Figure 1B showed that the native CaEno1 protein (around 50 kDa) was specifically recognized by IgY antibodies after the 7th immunization (lane 3) but not by those from preimmunized chickens (lane 2).

### 2.2. Construction and Panning of Anti-CaEno1 Antibody Libraries

Two antibody libraries containing scFv molecules with a short linker or a long linker were constructed. The sizes of these libraries were approximately 2.4 × 10^6^ and 1.36 × 10^7^ phage clones, respectively (Table 1). A 30-fold increase in the eluted titers of the library with a short linker was observed after the 4th round (Table 1), suggesting that the clones with specific binding affinity were enriched throughout the panning process. However, this phenomenon was not observed when the antibody library with a long linker was applied. The nucleotide sequences of 10 clones randomly selected from the library with a short linker were determined and aligned with the chicken immunoglobulin germline gene. The results indicated that identical heavy and light variable genes (V_H_ and V_L_) were used for scFv expression in all analyzed clones. This particular clone was labeled CaS1.

### 2.3. Binding Specificity of CaS1 scFv

The CaS1 scFv antibody was purified (Supplementary Figure S2) and its binding specificity to CaEno1 was determined by SPR analysis (Supplementary Figure S3). The association and dissociation rate constants were calculated as 9.83 × 10^4^ M^−1^s^−1^ and 1.91 × 10^−3^ s^−1^, respectively, resulting in an equilibrium dissociation constant value (*K*_D_) of 1.94 × 10^−8^ M.

### 2.4. Attenuation of C. albicans Growth by CaS1

**Figure 2:**
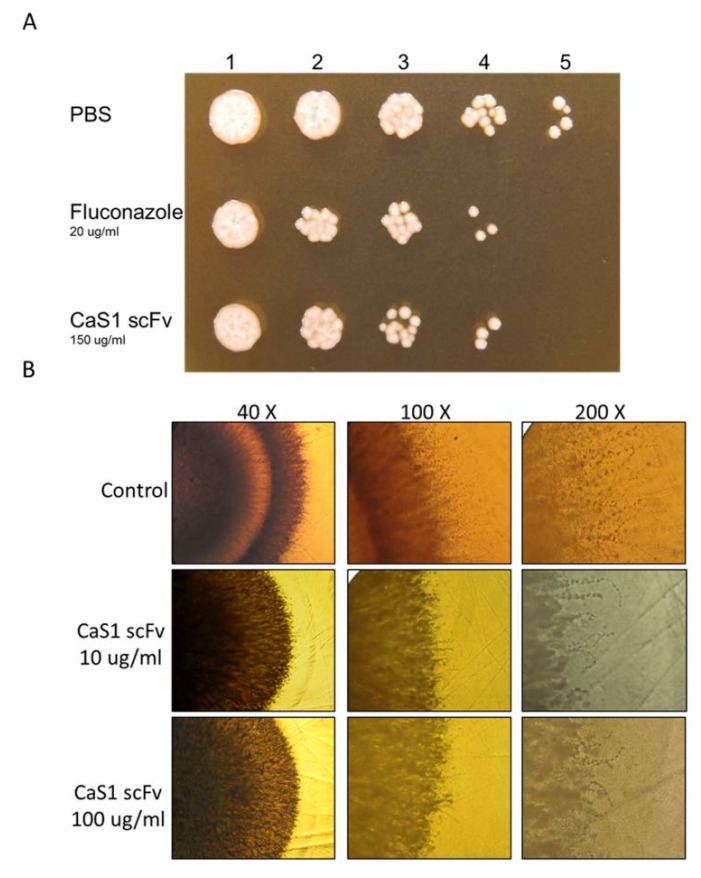
The inhibitory effects of the CaS1 single chain variable fragment (scFv) antibody. *C. albicans* (SC5314) with 5-fold serial dilution from 5^−1^–5^−5^ were (#1–#5) mixed with equal volumes of 20 μg/mL fluconazole, 150 μg/mL CaS1 scFv or PBS was spotted onto YPD agar plates as described in the text (**A**). *C. albicans* mixed with PBS, 10 or 100 μg/mL CaS1 scFv was spotted onto YPD (**B**). The hyphal formation was observed and recorded under a microscope with 40×, 100×, and 200× magnification. Presented data is representative of at least three independent experiments.

As shown in Figure 2A, CaS1 scFv attenuated *C. albicans* growth as effectively as treatment with fluconazole. Notably, the inhibitory effect of CaS1 was comparable to that of the polyclonal anti-CaEno1 IgY. After incubating CaS1 with *C. albicans,* the hyphae growing outwards from the fungus spot was investigated under microscopy. Hyphae were clearly visualized along the edge of the colony treated with 1× PBS, whereas less abundant hairy hyphae were observed in the CaS1-treated colonies (Figure 2B).

### 2.5. Binding of CaS1 to Eno1 Proteins of Different Candida spp.

**Figure 3:**
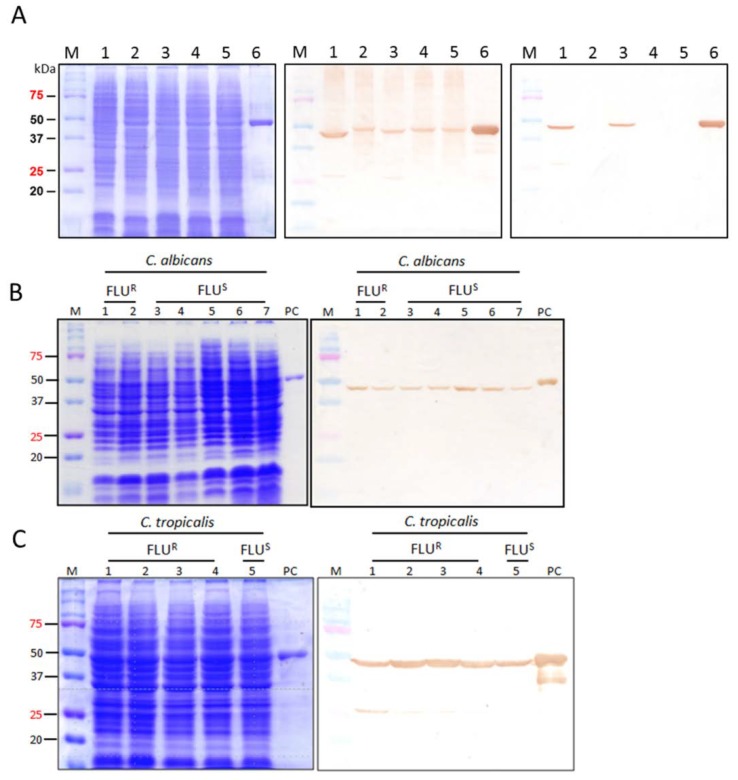
Binding specificity of CaS1 scFv. Total cell lysates of five *Candida* spp. were visualized by SDS-PAGE (**A**, left) and probed with purified IgY antibodies from chickens after the 7th immunization (1:3000) (**A**, middle) or CaS1 scFv (**A**, right). Lanes 1–6 contained the total cell lysates of *C. albicans* SC5314, *C. krusei* (clinical isolate), *C. tropicalis* (BCRC 20520), *C. parapsilosis* (BCRC 20515), *C. glabrata* (BCRC 20586) and purified rCaEno1. Similarly, total cell lysates of various strains of fluconazole-resistant (FLU^R^) and fluconazole-susceptible (FLU^S^) *C. albicans* (**B**) and *C. tropicalis* (**C**) were visualized by SDS-PAGE (left) and probed with purified CaS1 scFv (right). Lanes 1–7 in panel B contained the total cell lysates of *C. albicans,* including 2 FLU^R^ (CA6-17, CA7-26) and 5 FLU^S^ (CA7-3, CA10-50, CA7-30, CA10-65, SC5314) strains. Lanes 1–5 in panel C contained the total cell lysates of *C. tropicalis,* including 4 FLU^R^ (CT 6-29, CT11-52, CT8-63, CT6-50) and 1 FLU^S^ (CT12-54).

Purified CaS1 scFv was evaluated for its binding activity to Eno1 proteins of *C. albicans* SC5314, *Candida krusei*, *Candida tropicalis*, *Candida parapsilosis*, and *Candida glabrata*. Total cellular lysates were visualized by SDS-PAGE (Figure 3A, panel left). Anti-CaEno1 IgY specifically recognized the Eno1 of all five species of *Candida* (middle, lanes 1–5) as well as the rCaEno1 (middle, lane 6). Intriguingly, CaS1 only reacted with the Eno1 of *C. albicans* and *C. tropicalis* (right, lanes 1 and 3) but not those of *C. krusei* (lane 2), *C. parapsilosis* (lane 4), and *C. glabrata* (lane 5). Considering the high homology between Eno1 protein of *C. albicans* and *C. tropicalis*, the results suggested that a common antigenic epitope was shared by these 2 species but not by the others.

The binding activity of CaS1 to fluconazole-resistant and fluconazole-susceptible strains of four *Candida* spp. was examined. Their anti-microbial characteristics were confirmed by chromogenic *Candida* agar and listed in Supplementary Table S1. The representative results showed that CaS1 consistently recognized the CaEno1 protein of fluconazole-resistant and fluconazole-susceptible *C. albicans* (Figure 3B) and *C. tropicalis* (Figure 3C) but not that of *C. glabrata* and *C. parapsilosis* (Supplementary Figure S4).

### 2.6. Inhibitory Binding of the CaEno1 of C. albicans to Plasminogen by CaS1

**Figure 4:**
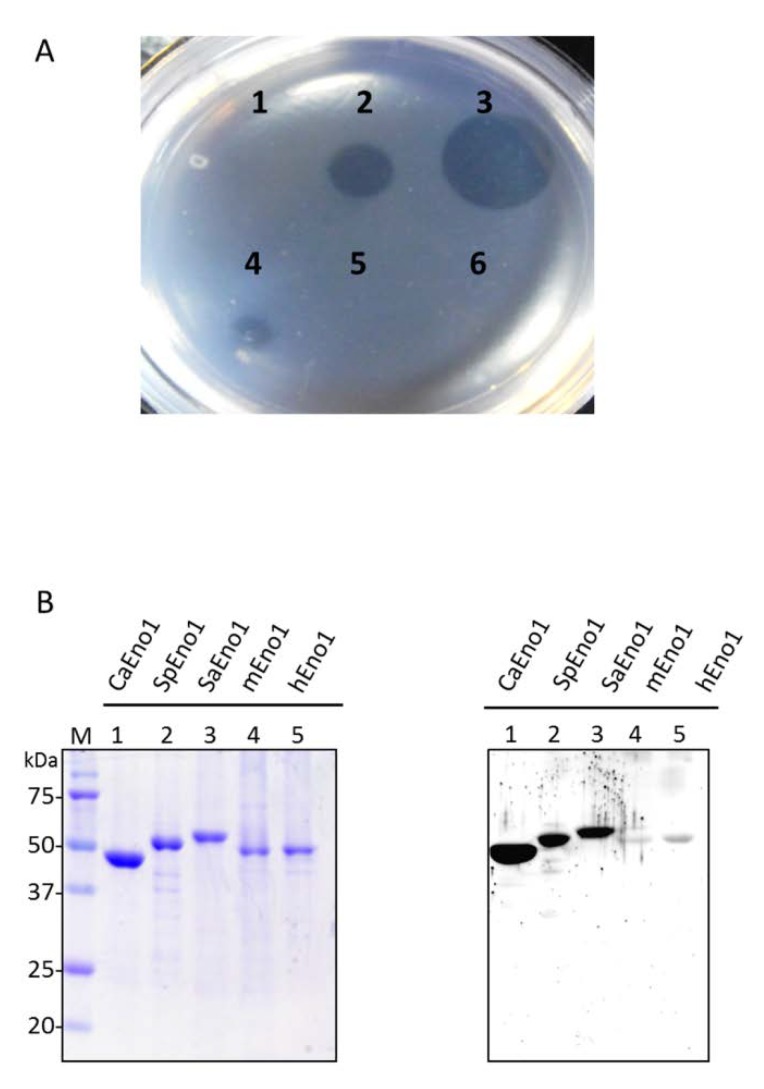
The effect of CaS1 scFv on the binding of Eno1 to plasminogen and the binding of different species of Eno1. The ability of CaS1 scFv to block the binding of CaEno1 to plasminogen was evaluated by fibrin matrix-gel degradation analysis. Various samples containing *C. albicans* only (**A**, 1), *C. albicans* + 1 µg of plasminogen (**A**, 2) or *C. albicans* + 10 µg of plasminogen (**A**, 3) were spotted onto the plate. Similar experiments were carried out, except that *C. albicans* was mixed first with 10 µg (**A**, 4) or 100 µg (**A**, 5) of CaS1 scFv, followed by the addition of 10 µg of plasminogen. A mixture of *C. albicans* and CaS1 scFv without plasminogen (**A**, 6) was spotted as a negative control. (**B**) The purified Eno1 proteins of *C. albicans*, *S. pneumonia*, *S. aureus*, mouse and human were visualized on SDS-PAGE (left). After transferred onto the NC (nitrocellulose) membranes, they were probed with purified CaS1 scFv (right) as described. Lane M contained protein markers. All data represent at least three independent experiments.

It is well known that surface Eno1 acts as a plasminogen receptor [16], which binding to plasminogen will lead to the activation of plasmin and subsequently the degradation of the fibrinogen (extracellular matrix). Study also suggested that CaEno1 is a major participant in systemic candidiasis [23]. We performed matrix-gel studies to test the possible inhibitory effect of CaS1 on the interaction between CaEno1 and plasminogen. No fibrinolytic activity was detected in the groups containing *C. albicans* only (Figure 4A1) or CaS1 only (Figure 4A6) in the absence of plasminogen. In contrast, fibrinolytic activity was significantly detected in the groups of *C. albicans* incubated with 1 and 10 μg of plasminogen (Figure 4A2,3). These results suggested that the plasminogen reaction with CaEno1 was activated by thrombin in the matrix gel, causing the degradation of the surrounding fibrinogen. Notably, the effect of fribrinolysis was dramatically suppressed by incubating *C. albicans* with CaS1 (10 or 100 μg), leading to the diminishing diameter of the plaques in a dose-dependent manner (Figure 4A4,5).

Next, we further examined whether CaS1 scFv would cross-react with Eno1 in other organisms. We purified the Eno1 from *S. pneumoniae* (SpEno1), *S. aureus* (SaEno1), mice (mEno1) and humans (hEno1). As shown by SDS-PAGE (left) and Western blot analysis (right), CaS1 scFv not only bound to CaEno1 but also cross-reacted with SpEno1 as well as SaEno1 but not with the Eno1 from mice and humans (Figure 4B).

### 2.7. Effects of CaS1 on the Survival of Mice and Zebrafish Infected with C. albicans

**Figure 5:**
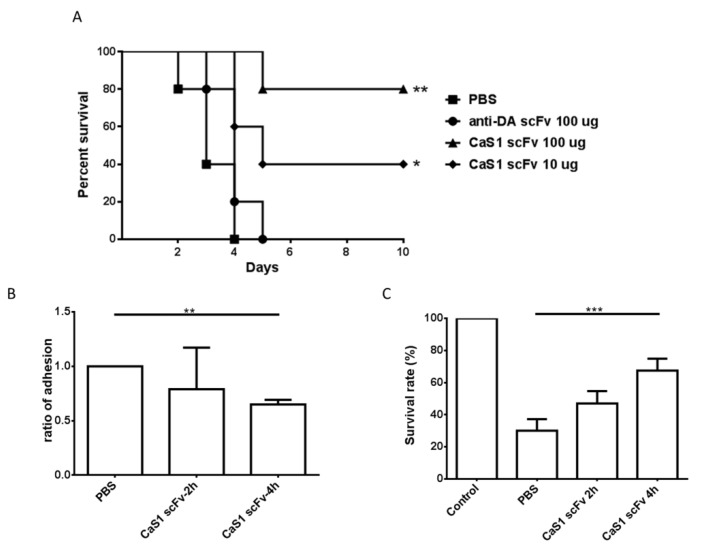
The effect of CaS1 scFv on the survival of mice and zebrafish models challenged with *C. albicans*. The mice were grouped and challenged with a mixture of 1 × 10^6^ *C. albicans* containing 10 µg CaS1, 100 µg CaS1, 100 μg anti-DA scFv (an unrelated scFv) or 1× PBS. (**A**) The survival of the mice was monitored at 1-day intervals for 10 days. The results showed that CaS1 scFv antibodies provide significant protection against the lethal challenge of *C. albicans* in mice (*n* = 5). (**B**) Twenty zebrafish embryos were co-incubated with 5 × 10^3^ cells/mL *C. albicans* in RPMI medium with/without 100 μg/mL CaS1 at 30 °C for 4 h. After co-incubation, non-adhesion *C. albicans* were removed by washing, and the embryos were incubated in egg water containing 0.5% YPD without or with 100 μg/mL CaS1 for an additional 2 days at 30 °C. These data were from two repeated experiments, and approximately 20 embryos were tested for each treatment. (**C**) The zebrafish embryo survival rate was calculated after co-incubation with *C. albicans* pretreated with CaS1 for 2 h and 4 h. Control indicated the embryos alone without *C. albicans*, PBS indicates *C. albicans* pretreated with PBS, CaS1 scFv 2 h refers to *C. albicans* pretreated with CaS1 for 2 h, and CaS1 scFv 4 h represents *C. albicans* pretreated with CaS1 for 4 h. The data were presented as the means ± SD. * *p* < 0.05, ** *p* < 0.01, *** *p* < 0.001.

Finally, we evaluated whether CaS1 scFv offers protection against *C. albicans* infection in mice. ICR mice were administered 1 × 10^6^ *C. albicans* cells mixed with CaS1 (10 or 100 μg) or an irrelevant anti-DA scFv (100 μg) via tail vein injection. All mice died after treatment with *C. albicans* or *C. albicans*/anti-DA *(Deinagkistrodon acutus snake)* scFv, while 40% to 80% of mice treated with the *C. albicans*/CaS1 remained alive (Figure 5A). Mice treated with CaS1 or fluconazole alone showed no effect on the survival rate. Taken together, these data suggest that CaS1 provides potential protection against lethal candidiasis in ICR mice.

We also performed this experiment using a zebrafish model, which has been increasingly applied in elucidating *C. albicans* pathogenesis [24]. The adhesion capability of *C. albicans* was inhibited by the CaS1 antibody. The relative adhesion capability of *C. albicans* pre-treated with CaS1 for 2 h and 4 h was reduced to 79% and 65%, respectively (Figure 5B). The data led to an increased survival time of zebrafish embryos in 2 h (47.6% vs. 30%, *p* = 0.08) and 4 h treated groups (67.5% vs. 30%, *p* = 0.0003) as compared to those of non-treated groups (Figure 5C).

### 2.8. CaS1 Reduced on Fungal Burden and Cytokine Levels on ICR Mice with Tail Vein Induced Candidiasis

**Figure 6:**
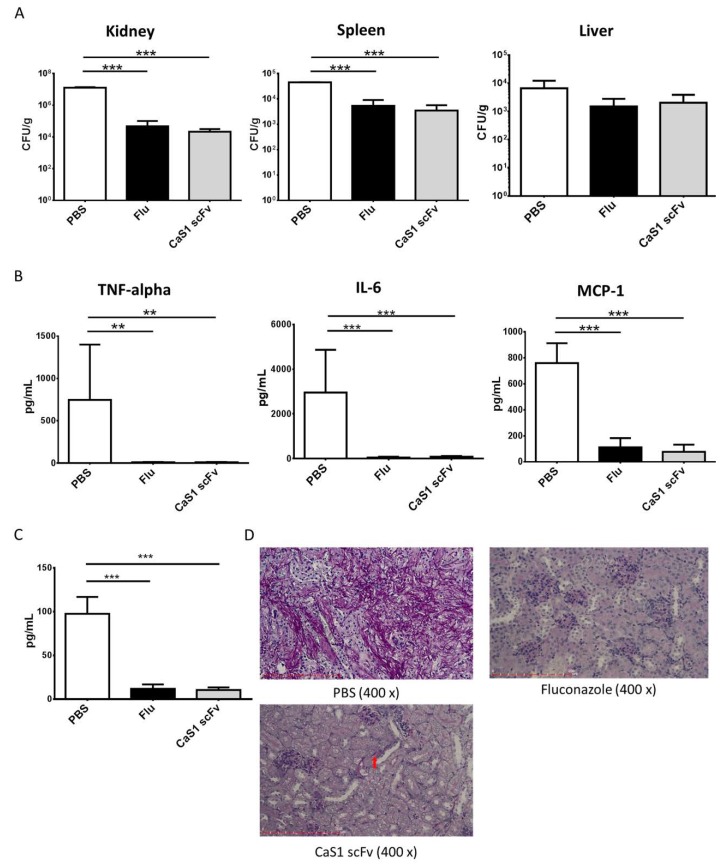
The effect of CaS1 scFv on fungal burden and cytokine levels in an ICR mouse model of candidiasis. Groups consisting of PBS-treated controls, fluconazole 5 mg/kg, or CaS1 scFv 8 mg/kg were tail vein injected at two hours after 1 × 10^6^ *C. albicans* SC5314 infection. (**A**) The kidney, spleen, and liver fungal burden were expressed as CFU/g (*n* = 5). (**B**) TNF-alpha, MCP-1 and IL-6 levels in mouse serum (*n* = 6–10). (**C**) The beta-d-glucan concentrations were measured in mouse serum. (*n* = 5). (**D**) PAS staining of kidney sections after different treatments. The arrows indicated *C. albicans* under the microscope investigation. The data were presented as the means ± SD. ** *p* < 0.01, *** *p* < 0.001.

For the in vivo model of infection, ICR mice were injected in the lateral tail veins with the 1 × 10^6^ CFU of *C. albicans* cells. After infected for 2 h, randomly grouped mice were tail veins injected with different treatment and sacrificed at 48 h as described in materials and methods. Fungal burden in the kidney (2.15 × 10^4^ ± 1.01 × 10^4^ CFU/g, *p* < 0.0001) and spleen (3493 ± 2161 CFU/g, *p* < 0.0001) but not the liver in CaS1 treated group was significantly reduced (Figure 6A). A reduction in fungal burden in the livers of mice treated with CaS1 (2036 ± 1850 CFU/g) or fluconazole (1503 ± 1289 CFU/g) was also observed than those of PBS-treated mice (6605 ± 5650 CFU/g), although the differences did not reach statistical significance. Analysis of blood samples showed CaS1 treatment significantly attenuated TNF-alpha (*p* = 0.0026), IL-6 (*p* < 0.0001) and MCP-1 (*p* = 0.0003) levels but did not affect IL-12p20, IFN-γ and IL-10 expression (Figure 6B). The mice injected with CaS1 or fluconazole alone without yeast infection showed no effect on fungal burden and cytokine levels. beta-d-glucan, a cell wall component of *Candida,* has been suggested as a biomarker of invasive fungal infections [25,26]. In Figure 6C, the results showed CaS1 or fluconazole significantly reduced beta-d-glucan concentrations. PAS staining showed much less *C. albicans* in kidney from CaS1- or fluconazole-treated mice compared with those from control mice (Figure 6D). Finally, relative ratio ΔCt *_C. albicans_*_–GAPDH_ (Supplementary Table S2) showed *C. albicans* in kidney were abundantly present in PBS-treated (group A), but less in CaS1-treated (group B), and not present in fluconazole-treated (group C) and non-infected mice (group D).

## 3. Discussion

*Candida albicans*, the opportunistic pathogen, is one of the leading causes of fungal infections in humans, especially in immunocompromised patients. Candidal diseases are often chronic, difficult to treat, and carry a high mortality and morbidity, despite antifungal therapy. Invasive candidiasis is a promising area for monoclonal antibody therapy because current therapies are inadequate [27,28,29]. Phage display antibody library methodology represents an excellent tool for the isolation of antibodies against the target. The enormous advances in the field allow the identification of relevant targets closely related to the clinical course of infectious disease. In this study, we identified a CaS1 scFv mAb by phage display, and the high specificity and binding activity of CaEno1, and its ability to attenuate cell growth, hyphal formation, and plasminogen binding may be further developed as a therapeutic.

Here, we evaluated the CaS1 scFv mAb in vitro and in vivo. CaS1 scFv bound only to fluconazole-resistant and fluconazole-susceptible *C. albicans* and *C. tropicalis* but not to the other *Candida* spp. tested (Figure 3C). *C. albicans* is responsible for the majority of Candida infections; however, infections by other less drug-susceptible *Candida* species have become more common recently [6,30]. Among them, *C. tropicalis* is predominantly identified in patients with leukemia and neutropenia [7,31]. As reported in the investigation of hospitalized patients with candidemia in China, *C. tropicalis* was the most commonly isolated *Candida* species in Nanjing [32] and the second most common in Shanghai [33]. In this context, CaS1 scFv demonstrated great value in the treatment against infections of *C. albicans* and *C. tropicalis*. Importantly, because the incidence of *Candida* spp. with resistance to fluconazole has dramatically increased, we believe that CaS1 scFv may provide an alternative treatment for patients infected with drug-resistant strains. In addition, CaS1 scFv bind not only to CaEno1 but also cross-react with SpEno1 as well as SaEno1 (Figure 4B). The cross-reactivity with *S. pneumoniae* and *S. aureus*, may due to the CaS1 scFv binding to similar epitope among these species. This effect may extend the usage of CaS1 scFv or may have combinatory effect in the clinical treatments for *S. pneumoniae* and/or *S. aureus* infections. A little or lack cross-reactivity of CaS1 scFv to human Eno1 will provide a great value for the possible treatment in human candidemia.

In both mouse and zebrafish models, treatment of infected mice with CaS1 scFv significantly improved the clinical outcome and clearance of *C. albicans* from the kidneys and spleen (Figure 6A). Studies have shown that an anti-enolase antibody inhibited the direct interaction of surface enolase with specific epithelial receptors on mouse intestinal disks, thus inhibiting *C. albicans* adhesion. Therefore, the extracellular enolase of *C. albicans* was involved in the colonization of mammalian intestinal epithelium [22]. The levels of the inflammatory cytokines and beta-d-glucan were significantly reduced in the CaS1 scFv-treated group. Therefore, these data suggest that CaS1 scFv may reduce the invasiveness of the infection caused by *C. albicans*.

In summary, we performed the isolation and characterization of a novel CaS1 scFv mAbs against CaEno1 from a phage display library in our present study. CaS1 scFv showed a lack of cross-reactivity with human enolases, and thus can further conjugate with antifungal small molecules to enhance the antifungal specificity, thereby diminishing or eliminating toxicity for the host. These results not only confirm that enolase plays an important role in invasive candidiasis but also show that CaS1 may be potentially useful for the development of immunotherapeutic agents against candidiasis.

## 4. Materials and Methods

### 4.1. Expression and Purification of his-CaEno1 Protein

The gene encoding CaEno1 was cloned into the pQE30 plasmid and transformed into *E. coli* BL21 cells. Recombinant his-CaEno1 protein was induced to express using isopropyl-β-d-thiogalactopyranoside (IPTG) and purified as described previously [34].

### 4.2. Animal Immunization

Female white leghorn (*Gallus domesticus*) chickens were first immunized with 50 μg of purified his-CaEno1 emulsified with Complete Freund’s adjuvant intramuscularly at different sites on the thighs. Then, his-CaEno1 mixed with Incomplete Freund’s adjuvant was given at intervals of 7 days. Polyclonal IgY antibodies were purified by using dextran sulfate [35,36]. ELISA was applied to determine the level of the humoral anti-his-CaEno1 immune response.

### 4.3. Construction and Biopanning of scFv Antibody Libraries

The antibody libraries were constructed by phage display as described [34]. In brief, spleens were placed in Trizol solution for homogenization. Total RNA (10 μg) was reverse transcribed into first-strand cDNA using the SuperScript RT Kit (Invitrogen, Carlsbad, CA, USA). Then cDNA template was amplified. In first round of PCR, immunoglobulin light chain (V_L_) and heavy chain (V_H_) fragments were amplified using specific chicken primers (Supplementary Table S3). During PCR process, the CSCVHo-F and CSCG-B primers were used for V_H_ with short linker (V_H-S_), the CSCVHo-FL and CSCG-B primers were used for V_H_ with long linker (V_H-L_), and the CSCVK and CKJo-B primers were used for V_L_. In each PCR reaction, the following was added into a 0.2 mL tube: 60 pmole forward and reverse primer, 1 μL synthesized cDNA, 50 μL 2× Taq DNA Polymerase Master Mix and adjusted the final volume to 100 μL. The PCR condition was performed under the following conditions: 94 °C for 5 min, then repeated 30 cycles of 94 °C for 15 s, 56 °C for 15 s and 72 °C for 90 s. The last step was incubated at 72 °C for 10 min. The PCR products were analyzed on 1% agarose gel and purified by gel extraction. In the second round of PCR, the scFv gene was generated. The following protocol was followed to amplify the full-length *scFv* gene, the CSC-F and CSC-B primers were mixed with the appropriate first-round purified PCR products. In each PCR reaction, the following was added into a new 0.2 mL tube: 60 pmole forward and reverse primer, 100 ng V_H-S_ or V_H-L_ products, 100 ng V_L_ products, 50 μL 2× Taq DNA Polymerase Master Mix and adjusted the final volume to 100 μL. The PCR condition was performed under the following conditions: 94 °C for 5 min, then repeated 25 cycles of 94 °C for 15 s, 56 °C for 15 s and 72 °C for 2 min. The last step was incubated at 72 °C for 10 min. The PCR products were analyzed on 1% agarose gel and purified by gel extraction. Then the purified scFv PCR products and pComb3X vector DNA were digested using SfiI (New England Biolabs, Ipswich, CA, USA) restriction enzyme. In each scFv digestion reaction, the following was added into a new 1.5 mL tube: 10 μg purified scFv-S or scFv-L, 360 units SfiI (36 units per μg of scFv DNA), 20 μL 10× cut-smart buffer and water was added to a final volume of 200 μL. In pComb3X vector digestion reaction, the following was added into a new 1.5 mL tube: 20 μg purified pComb3X DNA, 120 units SfiI (6 units per μg of pComb3X DNA), 20 μL 10× cut-smart buffer and water was added to a final volume of 200 μL. The mixtures were incubated for 5 h at 50 °C. The scFv products were purified using 2% agarose gel, and the pComb3X vector DNA was purified using 1% agarose gel through gel extraction. In ligation reaction, the following was added into a new 1.5 mL tube: 1.4 μg SfiI-digested and purified pComb3X, 700 ng SfiI-digested and purified scFv-S or scFv-L, 40 μL 5× ligase buffer and 10 μL ligase (Invitrogen by Thermo Fisher Scientific, Waltham, MA, USA) were added and adjusted the final volume to 200 μL using H_2_O. The mixtures were incubated for overnight at 23 °C. Then the ligated scFv full-length gene to pComb3X vector, which was also known as recombinant phagemid DNA, was ready for subsequent use. Recombinant phagemid DNAs were transformed into the *E. coli* ER2738 strain by electroporation (MicroPulser, Bio-Rad, Hercules, CA, USA). The recombinant phages were produced by the addition of wild-type VCS-M13 helper phage. Then, 10^11^ plaque-forming units (pfu) of recombinant phages in the scFv antibody libraries were added to wells coated with purified his-CaEno1 protein (0.5 μg/well). After removing the unbound phages, the remaining phages were used to infect the *E. coli* ER2738 strain. The amplified phages were recovered for the next round of selection as described above. After the 4th biopanning, total phagemid DNA from *E. coli* ER2738 was purified and transformed into *E. coli* TOP10F’. The expressed scFv antibodies were purified using Ni^2+^-charged sepharose (GE Healthcare Bio-Sciences AB, Uppsala, Sweden), which were further concentrated using Amicon Ultra-4 Centrifugal Filter Devices (Merck Millipore, Darmstadt, Germany) and examined for their binding or neutralizing capability against a panel of *Candida* spp., including *C. albicans* (SC 5314), *C. kruesi* (clinical isolate), *C. tropicalis* (BCRC 20520), *C. parapsilosis* (BCRC 20515) and *C. glabrata* (BCRC 20586).

### 4.4. C. albicans Growth and Hyphal Formation

To assess the effect of CaS1 scFv antibodies on cell growth, fluconazole (20 μg/mL) or CaS1 (150 μg/mL) was incubated with *C. albicans* (1 × 10^6^ CFU) at 37 °C for 1 h. After incubation, each mixture in 5-fold dilutions from 5^−1^–5^−5^ was spotted onto YPD agar plates and incubated at 37 °C overnight. To assess the effect on hyphal formation, CaS1 (10 or 100 μg/mL) was incubated with an equal volume of *C. albicans* (1 × 10^3^ CFU). Thereafter, each mixture was spotted onto YPD agar or Spider agar plates, followed by incubation at 37 °C for 5 days. For the invasion assay, YPD agar was flushed with PBS after incubating for 3 days. The hyphal invasion activity before and after flushing was compared.

### 4.5. Western Blotting

Purified his-CaEno1 protein or total cell lysates of five *Candida* spp. were transferred onto nitrocellulose membranes. The IgY (1:3000) from 7th-immunized chickens or purified CaS1 (1 μg/mL) was added and incubated. After washings, horseradish peroxidase (HRP)-conjugated donkey anti-chicken IgY antibodies (1:3000) (Jackson ImmunoResearch, West Grove, PA, USA) were added. However, goat anti-chicken light chain antibodies (1:3000) (Bethyl Laboratories, Montgomery, TX, USA), followed by the addition of HRP-conjugated donkey anti-goat IgG antibodies (Jackson ImmunoResearch, West Grove, PA, USA), were used to detect the bound CaS1 antibody. The proteins were visualized with diaminobenzidine (DAB) substrate.

### 4.6. Surface Plasmon Resonance (SPR)

Antigen/antibody binding interactions were analyzed using a localized surface plasmon resonance (LSPR) biosensor (OpenSPR, Nicoya Life Science Inc., Kitchener, Canada). CaEno1 was immobilized onto the carboxyl groups on the surface of sensor chips according to the manufacturer’s instructions. Briefly, 100 μL of CaEno1 (50 μg/mL) was introduced onto the sensor chip. Any remaining activated carboxyl groups were deactivated by treatment with OpenSPR deactivation buffer. During optimization experiments, CaS1 (100 μL, 30–65 μg/mL) was introduced onto a ligand-bound sensor chip. Sensorgram of the CaEno1/CaS1 interaction were recorded and analyzed using the built-in TraceDrawer software package (Ridgeview Instruments, Uppsala, Sweden).

### 4.7. Fibrin Matrix-Gel Degradation Analysis

The matrix gel was prepared to detect the fibrinolytic activity [17]. *C. albicans* (1 × 10^6^ CFU) were washed and incubated with 10 or 100 μg of CaS1. After incubation, the complexes were washed again and incubated with 10 μg of plasminogen for 30 min, which were then washed to remove free plasminogen. The resulting products were placed on a matrix gel containing 1.25% low-melting-temperature agarose, 0.05 U/mL thrombin and 2 mg/mL fibrinogen (Sigma, St. Louis, MO, USA). The gel was placed in a humidified chamber at 37 °C for 10–14 hrs. The level of fibrinolytic activity was correlated with the diameter of clear spots on the gel.

### 4.8. Effect of CaS1 on C. albicans Infection in Mice

For the survival rate analysis, ICR female mice (National Laboratory Animal Center, Taiwan) weighing approximately 25 g were randomly divided into 5 mice per group. Four groups of mice were injected through the lateral tail vein with the following preparations: (i) 1× PBS alone; (ii) 1 × 10^6^ CFU of *C. albicans* cells/10 μg of CaS1; (iii) 1 × 10^6^ CFU of *C. albicans* cells/100 μg of CaS1; and (iv) 1 × 10^6^ CFU of *C. albicans* cells/100 μg of anti-DA scFv. The mice were monitored for survival at one-day intervals for 10 days.

For the in vivo model of infection, ICR mice were injected in the lateral tail veins with the 1 × 10^6^ CFU of *C. albicans* cells. After infected for 2 h, randomly grouped mice were lateral tail veins injected with (A) PBS alone; (B) 8 mg/kg of CaS1; and (C) 5 mg/kg of fluconazole and sacrificed at 48 h. In parallel, mice without yeast infection were injected with (D) PBS; (E) CaS1 or (F) fluconazole, respectively. Tissues from kidneys, spleen and liver were aseptically removed, weighed and homogenized with 0.5 mL of PBS. Then, 200 μL of the homogenate was plated onto YPD plates overnight, which were counted for CFU. The fungal burden was expressed as a ratio of CFU/g of organ.

A mouse inflammation cytometric bead array (CBA) kit and flow cytometry with CBA v1.4 software (BD Bioscience, San Jose, CA, USA) were used to measure the cytokine levels according to the manufacturer’s protocol. (1,3)-beta-d-glucan in serum was detected using Glucatell kits (Associations of Cape Cod, South Yarmouth, MA, USA). The paraffin-embedded renal tissues were subjected to Periodic Acid-Schiff (PAS) staining. The presence of *C. albicans* in kidney was identified using the QuantStudio^TM^ 5 System (Thermo Fisher Scientific, Waltham, MA, USA). Briefly, SYBR green Master Mix, genomic DNA (100 ng/well) and primer specific for *C. albicans* (F: GGGTTTGCTTGAAAGACGGTA, R: TTGAAGATATACGTGGTGGACGTTA) were used for gene amplification [37]. The amplification conditions were 50 °C for 2 min; 95 °C for 2 min; 40 cycles of 1 s at 95 °C, and 30 s at 60 °C. The relative detection of *C. albicans* in tissue was adjusted with the presence of GAPDH.

### 4.9. Effect of CaS1 on C. albicans Infection in Zebrafish Model

Zebrafish (*Danio rerio*), aged approximately 12 months, were maintained in the zebrafish core facility at the National Health Research Institutes (NHRI) (http://www.zebrafishnthu-nhri.org/tzcf/) at 28 °C in a cycle of 10 h dark/14 h light [38]. Embryos were obtained from natural mating and staged as described [39]. One day post-fertilization, the embryos were sterilized using 0.002% chlorine bleach containing 0.0017% sodium hypochlorite to avoid potential contaminations.

### 4.10. Zebrafish Egg Bath Infection

Zebrafish egg bath infections were conducted according to established procedures [24]. In brief, wild-type *C. albicans* were grown with shaking in YPD plates at 30 °C overnight. Approximately 5 × 10^5^ cells/mL *C. albicans* in the presence or absence of 100 μg/mL CaS1 were incubated at 25 °C for 2 h or 4 h. Twenty embryos were co-incubated with 5 × 10^3^ cells/mL *C. albicans* in RPMI medium or RPMI medium containing 100 μg/mL CaS1 at 30 °C for 4 h. Thereafter, non-adhesive *C. albicans* were removed by 3 times washing with egg water (2% sea salts). The embryos were incubated in egg water containing 0.5% YPD with or without 100 μg/mL CaS1 at 30 °C for one additional day. After removing the non-adhesive *C. albicans*, 20 embryos were minced by a high-speed vortex in a tube containing 1 mL egg water and 30 beads. 100 μL of the cell suspension was plated onto YPD agar plates, and colonies were counted after overnight culture. The adhesive ability of *C. albicans* treated with PBS only was defined as 1 to estimate the inhibitory effects of CaS1.

### 4.11. Statistical Analysis

For survival curve analysis, the statistical significance of the differences in frequencies and proportions was determined by the log-rank test. The differences between the two groups were calculated using Student’s *t-*test (GraphPad Prism 6). The data are presented as the means ± SD, and a *p* value < 0.05 was considered significant. * *p* < 0.05, ** *p* < 0.01, *** *p* < 0.001.

### 4.12. Ethics Statement

Studies involving chickens and mice were approved by the Institutional Animal Care and Use Committee of Taipei Medical University (IACUC Approval No: LAC-2015-0302 valid on 1 February 2016 to 31 July 2019, and LAC-2015-0303 valid on 1 June 2016 to 31 May 2018). Verification of mice studies and zebrafish studies were carried out according to the protocols approved by NHRI in Taiwan (NHRI-IACUC-106095-A and NHRI-IACUC-106092-A valid on 1 August 2017 to 31 July 2020).

## Figures and Tables

**Table 1:** The anti-CaEno1 library size and eluted phage titers after each round of panning.

Library	Linker Length *	Library Size	Eluted Phage Titers after Each Round of Panning			
			1st	2nd	3rd	4th
CaEno1-S	7 aa	2.4 × 10^6^	9.6 × 10^4^	9.0 × 10^5^	7.2 × 10^5^	3.0 × 10^6^
CaEno1-L	18 aa	1.36 × 10^7^	2.75 × 10^5^	1.2 × 10^5^	1.32 × 10^6^	1.2 × 10^5^

* Linker length of 7 aa and 18 aa are GQSSRSS and GQSSRSSGGGGSSGGGGS, respectively.

## Supplementary Materials

Supplementary materials can be found at https://www.mdpi.com/1422-0067/21/8/2903/s1.

## Abbreviations

Eno1	alpha-enolase
CaEno1	*C. albicans* alpha-enolase
SpEno1	*S. pneumoniae* alpha-enolase
SaEno1	*S. aureus* alpha-enolase
mEno1	mice alpha-enolase
hEno1	humans alpha-enolase
rCaEno1	recombinant *C. albicans* alpha-enolase
scFv	single chain variable fragment
AmB	Amphotericin
DA	*Deinagkistrodon acutus*
NC	nitrocellulose
MDR	multidrug resistance
V_H_	heavy variable genes
V_L_	light variable genes
*K*_D_	dissociation constant value
FLU^R^	fluconazole-resistant
FLU^S^	fluconazole-susceptible
h	hour
s	second
min	minute
