# Supplementary material for: Generation and Characterization of Single Chain Variable Fragment against Alpha-Enolase of Candida albicans
Source: Int J Mol Sci. 2020 Apr 21;21(8):2903. doi: 10.3390/ijms21082903 (PMC7215377; doi:10.3390/ijms21082903)
Supplement: Supplementary file 1 [file ijms-21-02903-s001.pdf]

Supplementary Data

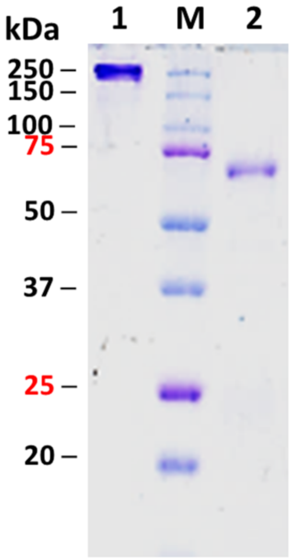

Figure S1. Analysis of purified polyclonal IgY antibodies. Adult female Leghorn chickens were immunized with purified CaEno1 protein for seven times at 7-day intervals. IgY antibodies purified from the eggs laid by immunized chickens were analyzed on Coomassie blue-stained SDS-PAGE under either non-reducing (lane 1) or reducing condition (lane 2). Approximately 50–75 mg of IgY antibodies could be obtained from each egg yolk.

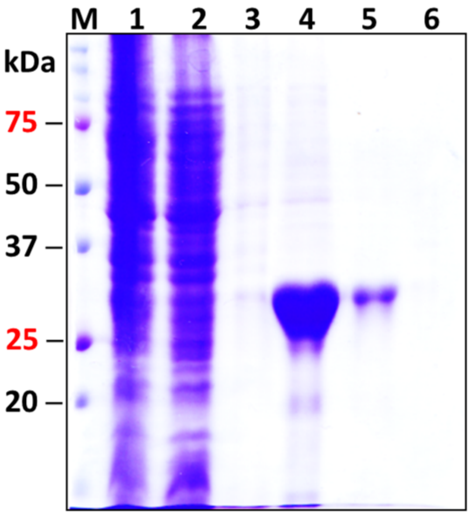

Figure S2. Expression and purification of CaS1 scFv antibody. After induced by IPTG, the *E. coli*-derived CaS1 scFv antibody was purified using Ni^{2+} sepharose and analyzed on Coomassie blue-stained SDS-PAGE. The molecular weight of CaS1 scFv was around 26 kDa as expected. Approximately 1.0–1.5 mg of CaS1 could be obtained from 1 L of bacterial culture. Lane 1: total cell lysate. Lane 2: flow through. Lane 3: washing fraction. Lane 4: elution 1. Lane 5: elution 2. Lane 6: Ni^{2+} sepharose.

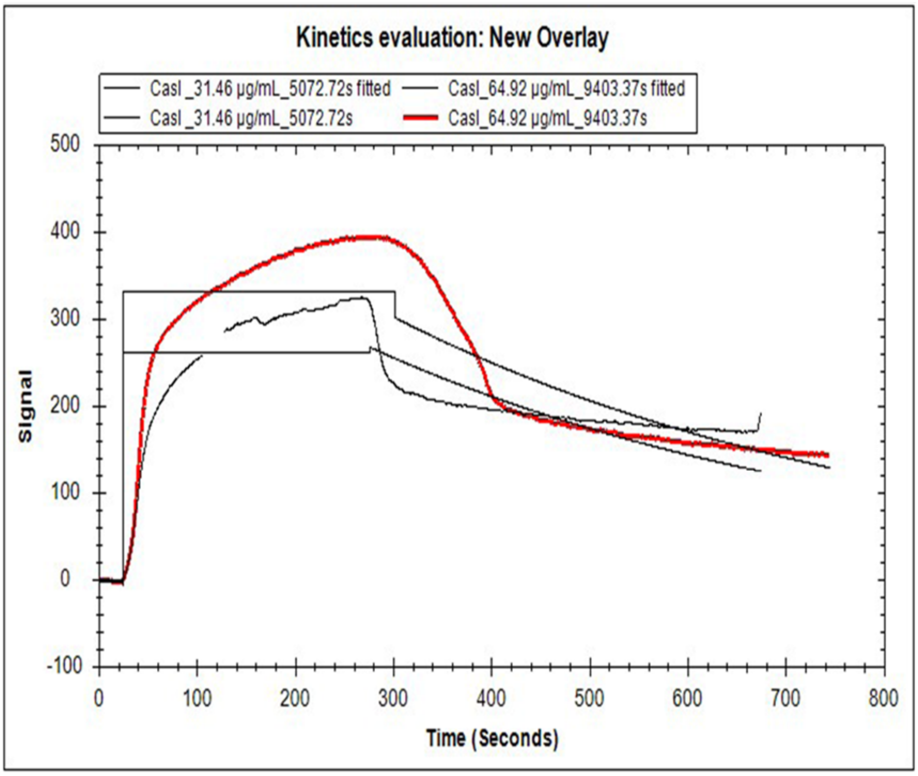

Evaluation type: OneToOne

Curve name	Bmax (Signal)	ka (1/(M*s))	kd (1/s)	KD (M)	BI (Signal)	Chi2 (Signal^2)
CasI _31.46 µg/mL_5072.72s fitted	267.33	9.83e4	1.91e-3	1.95e-8	-6.04	3088.68
CasI_64.92 µg/mL_9403.37s fitted	301.64	9.83e4	1.91e-3	1.95e-8	30.07	3088.68

Figure S3. Binding affinity of CaS1 determined by SPR method. The rCaEno1 was first immobilized on the sensor chips. Different concentrations of CaS1 (30–65 µg/mL) were then injected over the coated surface of sensor chip. Sensorgram of the CaEno1/CaS1 interaction was recorded and analyzed using the built-in TraceDrawer software package.

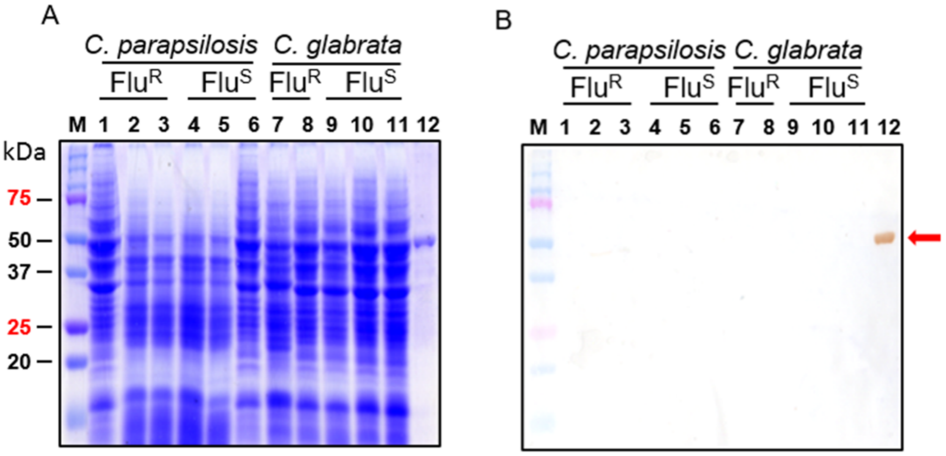

Figure S4. Binding analysis of CaS1 scFv against Eno1 protein expressed by *C. parapsilosis* and *C. glabrate*. Total cell lysates of five *C. parapsilosis* and four *C. glabrate* were visualized by SDS-PAGE (**A**) and probed with CaS1 scFv (**B**) as described in the text. Lanes 1–6 contained the total cell lysates of 3 FLU^R (CP 8-20, CP 12-27, CP 6-30) and 3 FLU^S (CP 7-17, CP 8-48, BCRC 20515) *C. parapsilosis* strains, respectively. Lanes 7–11 contained total cell lysates of 2 FLU^R (CG 5-8, CG 8-11) and 3 FLU^S (CG 7-37, CG 5-66, BCRC 20586) *C. glabrate* strains, respectively. Lane 12 contained rCaEno1 as a positive control (red arrow).

Table S1. Clinical fluconazole resistant and susceptible *Candida spp*. and their MIC.

Specimen	Organism	Drug	MIC (μg/mL)	Interpretation *
CA6-17	*C. albicans*	fluconazole	16	R
CA7-26	*C. albicans*	fluconazole	8	R
CA7-3	*C. albicans*	fluconazole	2	S
CA10-50	*C. albicans*	fluconazole	2	S
CA7-30	*C. albicans*	fluconazole	1	S
CA10-65	*C. albicans*	fluconazole	1	S
CT6-29	*C. tropicalis*	fluconazole	32	R
CT11-52	*C. tropicalis*	fluconazole	32	R
CT6-50	*C. tropicalis*	fluconazole	8	R
CT12-54	*C. tropicalis*	fluconazole	8	R
CG5-8	*C. glabrate*	fluconazole	64	R
CG8-11	*C. glabrate*	fluconazole	64	R
CG7-37	*C. glabrate*	fluconazole	32	S
CG5-66	*C. glabrate*	fluconazole	16	S
CP8-20	*C. parapsilosis*	fluconazole	256	R
CP12-37	*C. parapsilosis*	fluconazole	256	R
CP6-20	*C. parapsilosis*	fluconazole	16	R
CP7-17	*C. parapsilosis*	fluconazole	8	S
CP8-48	*C. parapsilosis*	fluconazole	4	S

* R: resistance; S: susceptible.

Table S2. The threshold cycle (Ct) values of *C. albicans* in tissues by real-time PCR.

Group	*C. albicans* Ct	GAPDH Ct	ΔCt $_{C.\ albicans\text{–}GAPDH}$
A2	22.26 ± 0.16	30.13 ± 0.15	−7.87
A4	20.64 ± 0.26	28.78 ± 0.19	−8.14
B1	38.18 ± 0.35	30.14 ± 0.01	8.04
B2	37.21 ± 0.04	30.56 ± 0.06	6.65
B4	35.55 ± 0.01	29.06 ± 0.47	6.49
C2	-	29.37 ± 0.36	#
C4	-	31.67 ± 0.54	#
C5	-	28.97 ± 0.01	#
D2	-	28.65 ± 0.33	#
D4	-	28.61 ± 0.28	#
D5	-	28.52 ± 0.06	#

-: Undetectable; #: not available.

Table S3. Primers used in the amplification of V_H and V_L genes.

Primers	Nucleotide Sequences
CSCVHo-F	5′-GGTCAGTCCAGATCTTCCGCCGTGACGTTGGACGAG-3′
CSCVHo-FL	5′-GGTCAGTCCTCTAGATCTTCCGGCGGTGGTGGCAGCTCCGGTGGTG GCGGTTCCGCCGTGACGTTGGACGAG-3′
CSCG-B	5′-CTGGCCGGCCTGGCCACTAGTGGAGGAGACGATGACTTCGGTCC-3′
CSCVK	5′-GTGGCCCAGGCGGCCCTGACTCAGCCGTCCTCGGTGTC-3′
CKJo-B	5′-GGAAGATCTAGAGGACTGACCTAGGACGGTCAGG-3′
CSC-F	5′-GAGGAGGAGGAGGAGGAGGTGGCCCAGGCGGCCCTGACTCAG-3′
CSC-B	5′-GAGGAGGAGGAGGAGGAGGAGCTGGCCGGCCTGGCCACTAGTGGAGG-3′
